# Nab-Paclitaxel Induced Cystoid Macular Oedema: Case Report and Literature Review

**DOI:** 10.22336/rjo.2025.76

**Published:** 2025

**Authors:** Tejas Shivarthi, Sujithra Haridas, Keechilat Pavithran

**Affiliations:** 1 Amrita School of Medicine, Amrita Institute of Medical Sciences, Amrita Vishwa Vidyapeetham, Kochi, Kerala, India; 2 Department of Ophthalmology, Amrita Institute of Medical Sciences and Research Center, Amrita Vishwa Vidyapeetham, Kochi, Kerala, India; 3 Department of Medical Oncology, Amrita Institute of Medical Sciences and Research Center, Amrita Vishwa Vidyapeetham, Kochi, Kerala, India

**Keywords:** cystoid macular oedema, albumin-bound paclitaxel, nab-paclitaxel, Abraxane, pancreatic cancer, optical coherence tomography, CMO = Cystoid Macular Oedema, CK = Cytokeratin, SATB2 = Special AT-rich sequence-binding protein 2, CDX2 = Caudal-type homeobox 2, PET = Positron emission tomography, BCVA = Best corrected visual acuity, SD-OCT = Spectral Domain Optical Coherence Tomography, CRT = Central Retinal Thickness, FA = Fluorescein Angiography, NSAID = Non-Steroidal Anti-inflammatory Drugs, VEGF = Vascular Endothelial Growth Factor, CAI = Carbonic Anhydrase Inhibitor

## Abstract

**Introduction:**

Nab-paclitaxel, a nanoparticle formulation of paclitaxel, is commonly used to treat solid tumors but is associated with rare adverse effects, including cystoid macular oedema (CMO). This case highlights the CMO in a patient undergoing nab-paclitaxel treatment for metastatic pancreatic cancer.

**Methods:**

A 72-year-old male receiving nab-paclitaxel and gemcitabine presented with progressive bilateral vision loss. Ophthalmological evaluation, including spectral-domain optical coherence tomography (SD-OCT), confirmed bilateral CMO. Nab-paclitaxel was discontinued, and the chemotherapy regimen was adjusted accordingly.

**Results:**

Visual symptoms improved significantly within 1 month of nab-paclitaxel cessation. Follow-up OCT and best-corrected visual acuity (BCVA) assessments showed significant improvement.

**Discussion:**

In this case, nab-paclitaxel–induced cystoid macular oedema (CMO) was confirmed by OCT and resolved rapidly after drug cessation. Review of 32 cases revealed predominantly bilateral involvement with highly variable onset and recovery times. In pancreatic cancer patients (n=8), cessation was the main management, with symptom onset ranging from 1–6 months and recovery typically within weeks to months. The variability in presentation may reflect acute toxic effects on Müller cells, disrupting retinal fluid homeostasis. Cases treated with carbonic anhydrase inhibitors showed faster resolution, supporting their role in modulating subretinal fluid dynamics. Alternative therapies such as NSAIDs or anti-VEGF agents demonstrated limited benefit, highlighting a non-inflammatory pathogenesis.

**Conclusions:**

Nab-paclitaxel-induced CMO is a rare but reversible condition that requires prompt cessation. Carbonic anhydrase inhibitors or anti-VEGF agents may expedite recovery in refractory cases. This report underscores the importance of early recognition and management of ocular toxicity in patients receiving nab-paclitaxel.

## Introduction

Taxanes, derived from natural products, were introduced as antitumor agents and approved for the treatment of solid tumors. Paclitaxel, a mitosis-inhibiting anti-microtubule agent, is used as a first- or second-line therapy for various cancers, either alone or in combination with other drugs. It is most commonly indicated for non-small cell lung, breast, and ovarian cancers. Due to its high lipophilicity and poor water solubility, the first commercial formulation (Taxol®) included ethanol and polyethylene castor oil to improve cellular delivery [**1-3**].

Nanoparticle albumin-bound paclitaxel (nab-paclitaxel), a protein-bound nanoparticle version, offers more potent antitumor activity in solid tumors resistant to or relapsing after chemotherapy. Abraxane is the most widely used formulation [**2**]. However, systemic and ocular side effects still occur. The most frequent hematologic side effects are leukopenia and neutropenia (more common with docetaxel), while anemia is more associated with paclitaxel. Non-hematologic side effects include edema, peripheral neuropathy, skin reactions, and mucositis [**4,5**]. Edema is caused by fluid retention and reduced colloidal osmotic pressure.

Ocular side effects may affect both anterior and posterior segments of the eye, with dry eye, superficial keratopathy, and an angiographically silent form of cystoid macular oedema (CMO) being the most commonly reported [**6**]. CMO linked to paclitaxel was first described in 2007. It presents as normal choroidal and retinal vessel filling without leakage. Paclitaxel is believed to damage Müller and retinal pigment epithelial cells, leading to accumulation of intra- and extracellular fluid [**7**]. Nab-paclitaxel-induced CMO is rare, and its treatment is not well established. Cessation of the drug remains the only consistently effective intervention.

We present a case of nab-paclitaxel-induced CMO in an elderly male undergoing treatment for metastatic pancreatic cancer.

## Case report

A 72-year-old male, a known case of type 2 diabetes mellitus, systemic hypertension, dyslipidemia, and liver cirrhosis, was diagnosed with metastatic pancreatic cancer in June 2023. Immunohistochemical staining yielded strong positivity for CK7 and CK19, but negative for CK20, SATB2, CDX2, and hepatocyte markers. In view of stage IV disease, the patient was initiated on a chemotherapy regimen consisting of gemcitabine and nab-paclitaxel. Approximately 3.5 months after cycle 4, the whole-body PET scan showed a partial response to therapy. However, the patient had complained of progressive vision loss, accompanied by watering of both eyes and a foreign-body sensation, for approximately 1 month before presentation.

**Fig. 1:**
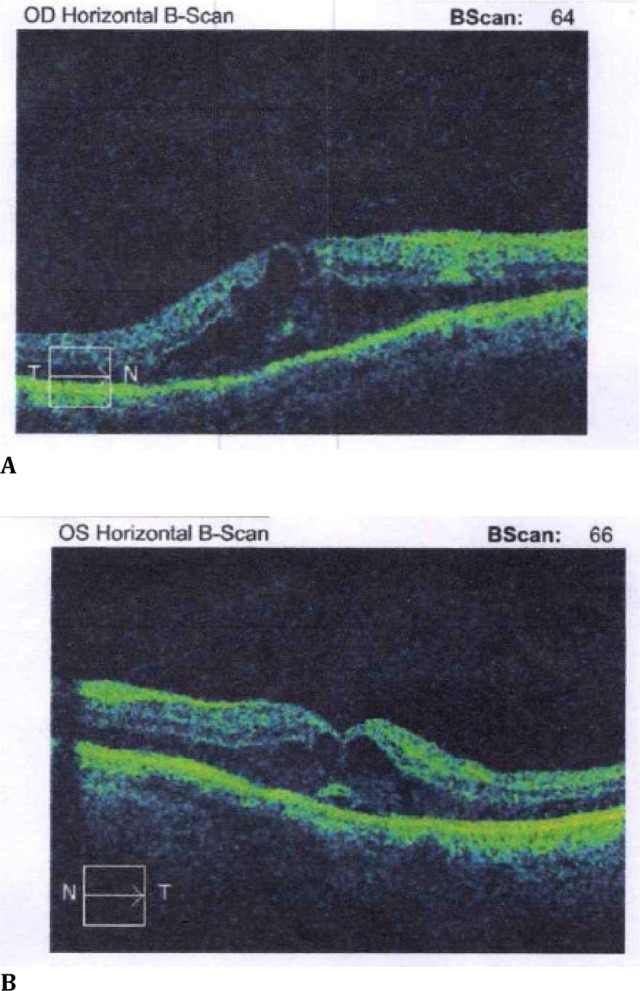
**A, B**. OCT revealing prominent macular oedema in both eyes at diagnosis

At the initial ophthalmologic examination, the best-corrected visual acuity (BCVA) was 6/18 in the right eye and 6/24 in the left eye. The anterior segment examination was unremarkable, and fundus fluorescein angiography was not performed due to the patient’s health status. Spectral-domain optical coherence tomography (SD-OCT) (Cirrus HD-OCT 4000; Carl Zeiss Meditec, Inc., USA) revealed retinal thickening with several intraretinal hyporeflective cystic honeycombed appearances in the outer plexiform layer of the retina, suggestive of cystoid macular oedema (**Fig. 1A, B**). The central retinal thickness (CRT) was -568 and 429 μm, in the right and left eyes, respectively. No other changes were evident on clinical examination or optical coherence tomography (OCT).

**Fig. 2:**
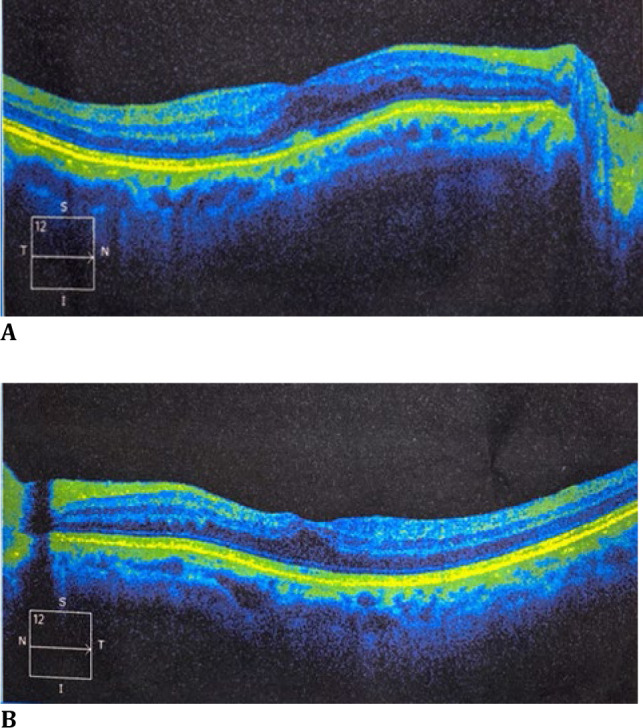
**A, B**. OCT revealing significant resolution of macular oedema after 1 month, nab-paclitaxel cessation

Nab-Paclitaxel was subsequently discontinued in view of the complaints, and the patient’s chemotherapy regimen was altered to gemcitabine and capecitabine. Following this, the patient showed significant improvement in his visual complaints at the 1-month follow-up examination, with BCVA of 6/9 in the right eye and 6/12 in the left eye. Dilated fundus examination revealed a normal optic disc and macula with normal vessels. OCT scans showed distortion in the foveal contour but no oedema in both eyes (**Fig. 2A, B**).

## Discussion

We confirmed our suspicion of macular oedema induced by nab-paclitaxel in this case based on the clinical presentation and OCT findings. Macular oedema had resolved entirely upon cessation of nab-paclitaxel. Macular oedema is a rare complication in patients treated with nab-paclitaxel that can cause a significant decline in visual acuity. A previously conducted phase III trial comparing nab-paclitaxel with polyethylated castor oil-based paclitaxel in the treatment of women with breast cancer reported visual disturbances in 13% of patients, with 1% classified as severe. Most visual changes were observed in patients receiving doses higher than the currently recommended 260 mg/m2 every 3 weeks [**8**]. Our patient received a dose of 125 mg/m2 every week at the time of treatment cessation. The time interval between exposure and clinical presentation is highly variable, ranging from a few months to approximately 2.5 years. Our patient had a gradual onset of visual loss one month before presentation. Progressive bilateral visual loss, as observed in our case, is a common complaint among patients due to cysts that thicken the parafoveal retinal layer [**6**]. The diagnosis is mainly confirmed by spectral-domain optical coherence tomography (SD-OCT) and fluorescein angiography (FA), which show fluid accumulation in the retina’s extracellular space, with increased involvement of the inner and outer nuclear layers, leading to abnormal macular thickening [**9**]. This was clearly observed in the patient’s OCT scan, thereby strengthening the diagnosis.

The complete resolution of macular oedema without drug cessation alone led us to believe that, in our case, the pathogenetic mechanism of CMO was similar to that suggested by Joshi and Garretson, implying a toxic effect of paclitaxel on Müller cells, which are responsible for osmotic gradient maintenance within the neurosensory retina, resulting in intracellular fluid accumulation [**7**].

A review of the existing literature yielded 32 published case reports of induced macular oedema secondary to nab-paclitaxel treatment for various cancers. In these patients, the male-to-female ratio was 1:4, with a mean age of 56.5 years. Eighteen female patients were diagnosed with metastatic breast cancer, with male patients being treated for melanoma, pancreatic, lung, and hypopharyngeal cancer. The vast majority of cases present with bilateral eye involvement, with the unilateral participation observed in only one case [**10**]. Alternative treatment options for macular oedema included dorzolamide in 6 cases (18.75%); however, most cases (n=15), including our own, adopted complete cessation of nab-paclitaxel as the sole management approach. Treatment cessation was observed in all cases except for one published by Meyer et al., which was the only case of paclitaxel-induced maculopathy effectively treated without paclitaxel discontinuation. The patient was treated with acetazolamide for eight weeks and was still receiving paclitaxel for two years following the initial episode of CMO [**11**].

We identified only eight published case reports, detailing an episode of macular oedema secondary to nab-paclitaxel treatment for the pancreas as the primary tumor site (**Table 1**). The patients, including our patient, had a mean age of 62.8 years. Three of the patients (33%) were females. Bilateral macular involvement was observed in all the cases. The dexamethasone intravitreal implant was a successful treatment approach in one patient (11.1%) [**12**], and topical dorzolamide therapy was used in another patient (11.1%). Cessation of nab-paclitaxel treatment was the definitive treatment in 7 cases (77.78%). The mean time to symptom onset in our case (1 month) was much shorter than that reported in studies by Burgos-Blasco et al. (6 months) and Lee et al. (4 months) [**13,14**]. As mentioned previously, nab-paclitaxel-induced CMO was highly variable in the duration of symptom onset, and we attributed this to the acute presentation observed in our case. Interestingly, our case showed the shortest time to recovery following drug cessation (1 month) compared with other cases of nab-paclitaxel used in the treatment of pancreatic cancer (1.15–6 months).

**Table 1:** Case reports of cystoid macular oedema in pancreatic cancer patients treated with albumin-bound paclitaxel

Author, year	Sex	Age (years)	Involved eye	Fluorescein Angiography	Treatment	Treatment time to recovery (months)
Ota et al., 2021	Male	71	Bilateral	-	Drug Discontinuation	2 months
Burgos-Blasco et al., 2020	Male	67	Bilateral	No capillary leakage in late frames	Drug discontinuation and Intravitreal Dexamethasone	4 months
Alves Pereira et al., 2022	Male	61	Bilateral	Not performed given the patient’s health status	Drug Discontinuation	5 weeks
Otsubo et al., 2021	Male	72	Bilateral	No retinal hemorrhage or exudates	Topical Dorzolamide	2 months
Junhyuck et al., 2019	Female	43	Bilateral	Absent or minimal late-phase leakage at the fovea	Drug Discontinuation	3 months
Sridhar et al., 2016	Female	48	Bilateral	-	Drug discontinuation	-
Ito et al., 2017	Female	73	Left eye	No pooling or leakage in the late phase	Drug discontinuation	6 months
Hussain et al., 2022	Male	61	Bilateral	No evidence of leakage or neovascularization	Drug discontinuation and topical dorzolamide	3 months
Present case, 2024	Male	72	Bilateral	Not performed given the patient’s health status	Drug discontinuation	1 month

In our case, CMO resolved with the cessation of nab-paclitaxel alone, the most widely adopted management modality, as observed in previous cases [**13-16**]. Based on the conclusions of Otsubo et al., treatment cessation was considered because of the marked resistance of the CMO to conventional treatment [**12**]. There are no established treatment guidelines for paclitaxel-induced CMO, and a review of the literature reveals heterogeneity in treatment options. In the analysis of all retrieved cases, the recovery period ranged from 3 weeks to 11 months, with a mean period of 2.31 months. The analysis of cases with no other treatment besides drug cessation showed a mean resolution time of 2.93 months. The recovery duration observed in the present study was shorter than the duration reported in this study. The analysis of cases treated with dorzolamide (DRZ) (the most commonly used drug) yielded a shorter mean resolution duration of 1.65 months (n=6). This significant decrease in resolution time suggests considering topical dorzolamide as part of the definitive treatment for paclitaxel-induced CMO. This is further supported by the findings of Ehlers et al., who reported a monocular control trial showing rapid resolution of CMO in the eye treated with topical carbonic anhydrase inhibitors, compared with the untreated eye after nab-paclitaxel cessation [**17**]. The use of acetazolamide in the case mentioned above by Meyer et al. yielded an excellent response, with complete resolution of oedema without cessation of nab-paclitaxel [**11**]. Another case reported by Ito et al. described the successful treatment of macular oedema with acetazolamide and cessation of paclitaxel [**18**].

Other treatment modalities employed the use of NSAIDs, which were shown to increase the mean recovery time to approximately 4 months and are hence considered ineffective. This also implies that the CMO is not primarily mediated by inflammation.

Three cases were identified in a review that employed anti-vascular endothelial growth factor (VEGF) treatment: two with intravitreal bevacizumab [**19,20**] and one with intravitreal ranibizumab [**21**]. Both treatments resulted in resolution within 2 months, with narrowing of the oedema area observed in the patient treated with intravitreal ranibizumab. The overall effectiveness, however, was limited, suggesting that anti-VEGF therapy played a minor role in the treatment outcome.

However, the underlying pathology of nab-paclitaxel remains unclear. As mentioned earlier, we proposed that the CMO in our case might have been attributed to disturbances of water channels in the retinal Müller cells and retinal pigment epithelium, caused by paclitaxel-mediated toxic effects on microtubules that disrupt fluid homeostasis. This might explain the resolution of macular oedema with immediate cessation of nab-paclitaxel observed in almost all reported cases. A previous study concluded that carbonic anhydrase (CA) XIV, an extracellular, membrane-bound CA identified in retinal Müller cells and the RPE, plays a role in extracellular pH homeostasis [**22**]. They proposed that CA XIV enhances subretinal fluid absorption in macular oedema, which would explain the shorter recovery time observed in CAI-treated patients.

## Conclusion

Our case highlights a rare presentation of nab-paclitaxel-induced macular oedema. A high degree of suspicion is maintained by clinicians encountering nab-paclitaxel-treated patients who present with progressive or sudden onset of visual complaints. Prompt cessation of treatment appears to be the only proven management approach, with a combination of carbonic anhydrase inhibitors or anti-VEGF agents considered in cases of long-standing CMO.

## Data Availability

Patient data and additional information will be made available by the corresponding author on reasonable request.
